# The Potential of panHER Inhibition in Cancer

**DOI:** 10.3389/fonc.2015.00002

**Published:** 2015-01-28

**Authors:** Xiaochun Wang, Kathleen M. Batty, Philip J. Crowe, David Goldstein, Jia-Lin Yang

**Affiliations:** ^1^Sarcoma Nano-Oncology Group, Adult Cancer Program, Lowy Cancer Research Centre, Prince of Wales Clinical School, University of New South Wales (UNSW), Sydney, NSW, Australia; ^2^Department of Surgery, Prince of Wales Clinical School, University of New South Wales (UNSW), Sydney, NSW, Australia; ^3^Department of Medical Oncology, Prince of Wales Clinical School, University of New South Wales (UNSW), Sydney, NSW, Australia

**Keywords:** targeted therapy, panHER inhibitors, drug resistance, HER signaling pathways, EGFR

## Abstract

**Purpose:** Hyper-activation of the HER (erbB) family receptors, HER 1-4, leads to up-regulation of the three vital signaling pathways: mitogen activated protein kinase, phosphoinositide 3-kinase/AKT, and Janus kinase/signal transducer and activator of transcription pathways. Blocking HER1/EGFR has a limited anticancer effect due to either secondary mutation e.g., T790M or by-pass signaling of other HER members. The emergence of an anti-panHER approach to blockade of these pathways as a cancer treatment may provide a solution to this resistance. This review aimed to provide an overview of the HER signaling pathways and their involvement in tumor progression and examine the current progress in panHER inhibition.

**Methods:** Recent literature associated with HER signaling pathways and panHER inhibition was reviewed through PubMed and Medline database, followed by critical comparison and analysis.

**Results:** Pre-clinical studies and clinical trials of panHER inhibitors show promising results, and the potential to improve patient outcomes in solid cancers.

**Conclusion:** The use of panHER inhibitors in cancers with HER-family hyper-activation, such as other epithelial cancers and sarcoma, is a new direction to research and has potential in clinical cancer therapy in the future.

## Introduction

Cancer continues to pose a great problem and burden on society despite new treatment options. In Australia, it is the most common cause of death and the leading cause of burden of disease (19%) (1). In 2007, the incidence of cancer was 485 cases per 100,000 people and the death rate was 176 per 100,000 people. The most common cancers are prostate cancer, bowel cancer, breast cancer, melanoma of skin, and lung cancer. While surgery, radiotherapy, and chemotherapy have led to major improvements in patient prognosis, newer treatments are needed to more effectively manage this disease in its advanced stage.

Over-expression of the human epidermal growth factor receptor (EGFR/HER1) pathway is a feature of many cancers and a potential therapeutic target. Early research suggested that blocking EGFR/HER1 by its specific inhibitor may have activity in some cancers through tyrosine kinase signaling inhibition (2). However, such a blockade can induce secondary mutation (T790M) (3) and, in addition, has no impact on the other tyrosine kinase receptors within the HER-family (HER2/4) (4). This potentially allows by-pass signaling pathways to remain active; a prime example being the Janus kinase/signal transducer and activator of transcription (JAK/STAT) pathway, which is normally regulated *via* HER members (5). Other resistance mechanism like c-MET amplification/over-expression may also weaken the effect of HER-family inhibition (6, 7).

Understanding the mechanism of each HER-family member, their signaling pathways and interactions among them will have a great impact on designing treatment approaches to conquer the resistance of EGFR/HER1 targeted therapy. This review has endeavored to provide an overview of the HER signaling pathways and their involvement in tumor progression and to examine the current progress in HER-family inhibition.

## HER-Family Members and Their Signaling Pathways

The HER signaling pathways are normally involved in regulation of cell growth and survival as well as adhesion, migration, differentiation, and other cellular responses. An understanding of these pathways is vital in appreciating the action of panHER inhibitors and anti-HER member antibodies. There are four members of the family, including EGFR/HER1, HER2, HER3, and HER4 (also called erbB-1, erbB-2, erbB-3, and erbB-4, respectively).

Hyper-activation of these receptors culminates with downstream up-regulation of the mitogen activated protein kinase (MAPK), phosphoinositide 3-kinase/AKT (PI3K/AKT), and JAK/STAT pathways (8). In cancer, these pathways are linked to many cellular processes including tumor progression, angiogenesis, metastatic spread, and inhibition of apoptosis (9). This hyper-activation may be due to over-expression of HER ligands, receptors or sustained activation of receptors, as summarized in Table 1.

**Table 1:** **HER ligands and receptors**.

Receptor homodimer/heterodimer	Ligands	Over-expression associated malignancies
EGFR (HER1)	EGF, TGF-α, AR, HB-EGF, EPG, EPR, BTC	NSCLC, breast, glioma, head and neck, bladder, kidney, soft tissue sarcoma
HER2/HER3	EPR, NRG1-α, NRG2-β	Breast
HER3	NRG1-β, NRG2-β	Breast, colon, gastric, prostate, other carcinomas, soft tissue sarcoma
HER4	HB-EGF, BTC, EPR, NRG1-β, NRG2-β, NRG4	Childhood medullo-blastoma
HER2/HER4	EGF, TGF-α, HB-EGF, EPR, BTC, NRG2-α, NRG3	

*EGF, epidermal growth factor; TGF, transforming growth factor; AR, amphiregulin; HB-EGF, heparin binding epidermal growth factor; EPG, epigen; EPR, epiregulin; BTC, betacellulin; NRG, neuregulin*.

**Figure 1:**
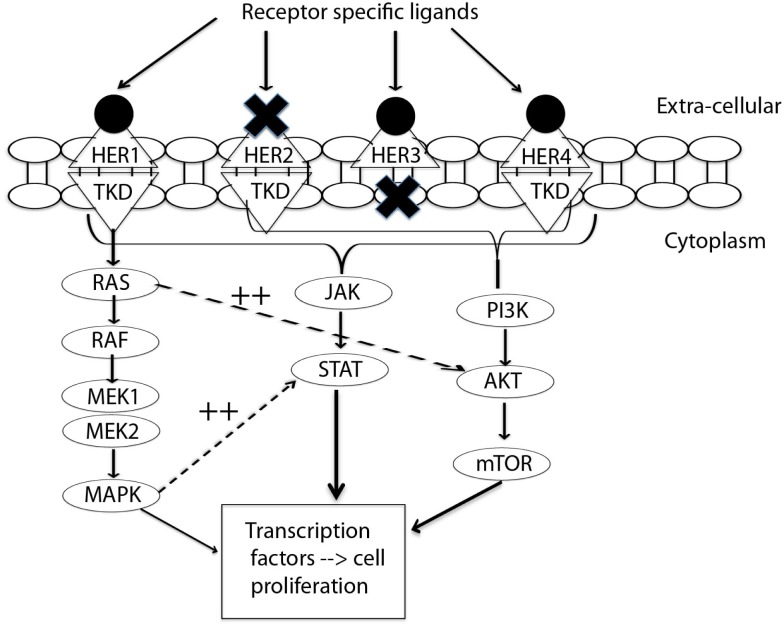
**Signal transduction by HER-family**. This figure summarizes the interplay between three pathways: MAPK, P13K/AKT, and JAK/STAT. MAPK dramatically enhances transcriptional activation by STAT (11). EGFR/HER1 cannot directly activate the P13K/AKT pathway (12), but it couples to the ras/MAPK pathway as well as to the ras/PI3K/AKT pathway (10). This interplay of pathways forms the source of by-pass resistance to EGFR TKIs. TKD, tyrosine kinase domain; MEK, mitogen activated protein kinase kinase; MAPK, mitogen activated protein kinase; PI3K, phosphoinositide 3-kinase; mTOR, mammalian target of rapamycin; JAK, janus kinase; STAT, signal transducer and activator of transcription.

The HER receptors are composed of a large extra-cellular ligand-binding domain, which has four subdomains (I–IV), followed by a transmembrane domain, a small intracellular juxtamembrane domain preceding the kinase domain, and a C-terminal tail, on which the docking sites for phosphotyrosine-binding effector molecules are found (10). These four receptors form homo-dimers and hetero-dimers, which are associated with instigation of different downstream pathways. Emerging targeted treatment has focused on inhibition of these HER receptors *via* either external monoclonal antibodies (mAb) or small molecule tyrosine kinase inhibitors (TKIs). Figure 1 presents a simplified summary of the HER signaling pathways.

### EGFR/HER1 pathways

EGFR/HER1 has been shown to be involved in proliferation and differentiation of epithelial tissues of the skin, lung, pancreas, and gastrointestinal tract (13). Binding of ligands to EGFR leads to auto-phosphorylation of critical tyrosine residues, which serve as attachment sites for various cellular-docking proteins to activate signaling cascades and affect gene transcription (14). Cellular-docking proteins include growth factor receptor bound-2 (GRB2) and Src-homology-2-containing (SHC), both concerned with recruitment of ras and activation of the MAPK cascades (15). Furthermore, MAPK specifically phosphorylates a serine near the C-terminus of most STATs, dramatically enhancing transcriptional activation by STAT (11).

EGFR/HER1 has highly specific recognition sites, and is unable to recruit PI3K (12). As such, EGFR/HER1 cannot directly activate the PI3K/AKT/protein kinase B (PKB) pathway, but it converges with the ras/MAPK pathway as well as *via* ras to the ras/PI3K/AKT/PKB pathway (10).

### HER2 pathways

HER2 only functions when partnered with either one of the other three type I receptor tyrosine kinase family members (HER1, HER3, or HER4) or type II receptor tyrosine kinase family members (IR or IGF-IR) (16–20). It is a current understanding that HER2 as an orphan receptor cannot directly bind any ligands. Rather, it dimerizes with ligand-bound receptors. However, when expressed as a heterodimer, the combination-receptor shows higher affinity and broader specificity for ligands than other heterodimer couples (21). These advantages are due to slower growth factor dissociation, as well as slow endocytosis of HER2 containing hetero-dimers. Hence, HER2-associated hetero-dimers show a strong proliferative potential as there is simultaneous and prolonged recruitment of multiple signaling pathways including the JAK/STAT pathway (22). Several types of cancer are associated with HER2 over-expression, being most thoroughly studied in breast cancer where there is gene amplification in 15–30% of invasive ductal carcinomas (23).

Trastuzumab is a humanized mAb directed against the extra-cellular domain of the HER2 receptor. It has been shown to have major clinical benefits when used in HER2 overexpressing breast cancer, which otherwise carries a very poor prognosis (24). The mechanism of action is complex, ultimately providing antibody-dependent cellular cytotoxicity (ADCC) against cells overexpressing HER2 as well as down-regulation of signaling pathways including MAPK and AKT (18).

In malignant tumors, constitutively activated HER2 and EGFR/HER1 not surprisingly stimulate many of the same intracellular signaling proteins and pathways as wild-type receptors, such as the MAPK, PI3K/AKT/mTOR pathway, Src kinase, and STAT transcription factors (25). However, differences arise due to impairment of the usual negative regulatory loops functioning in normal cells (26). Without such negative feedback, cells are able to proliferate in an uncontrolled manner.

### HER3 pathways

HER3 is another non-autonomous receptor, which forms functional hetero-dimers with the other members of the HER-family. It has defective kinase activity and only acquires signaling potential, that is, tyrosine phosphorylation, when dimerized with another receptor. Upon heterodimerization, the cytoplasmic domain of HER3 undergoes aforementioned tyrosine phosphorylation allowing recruitment of PI3K as well as SHC, although not GRB2 (27). Additionally, upon heterodimerization, HER3 strongly promotes PI3K activation; especially when binding to its’ preferred partner HER2 (28). Activation of PI3K pathway is closely associated with survival signals allowing avoidance of apoptosis (29).

HER3 is expressed in many cancers, although there is no evidence to suggest gene amplification and over-expression (30). In malignancies such as ovarian cancer where HER3 is frequently expressed (53.4%), expression has been associated with a shorter survival time (3.31 vs. 1.8 years median survival time: low HER3 expression and HER3 over-expression, respectively) (31).

### HER4 pathways

HER4 has many characteristics likening it to EGFR/HER1 including recruitment of GRB2, SHC, and STAT5. One isoform of HER4 is able to activate the PI3K/AKT pathway (32). HER4 tends to have low expression in breast and prostate cancers, yet over-expression is frequently seen in other cancers (e.g., >50% childhood medullo-blastomas) (33). In such cases, heterodimerization with HER2 is correlated with malignancy, suggesting increased autocrine or paracrine loop signaling involving Neuregulin 1 (NRG1) (34).

### HER pathway synergy

As referred to above, co-operation between HER receptors has been observed in oncogenic transformation, both *in vitro* in cultured cells and in primary human tumors. For example, HER3 expression increases HER2-mediated transformation and tumorigenic growth in NIH3T3 cells (35). Additionally, co-expression of EGFR/HER1 or HER2 is necessary for NRG-1-induced transformation of fibroblasts by HER4 (8). The enhanced proliferative activity of cells expressing multiple HER receptors are presumably due to the interaction and strength of signaling in HER receptor combinations (36). HER pathway synergy results in additional autocrine loops driven by co-expression of multiple HER proteins. Such pathways are closely linked with tumor progression (8). This synergy of multiple pathways also provides an “escape route” whereby blocking specific member of the HER-family results in functional compensation by parallel HER pathways. It follows that panHER inhibition may overcome this shortcoming of single targeted therapies. Table 2 summarizes the regulation mechanisms of the HER signaling pathways.

**Table 2:** **HER signaling regulation**.

Regulation mechanism	Effect on signaling	Mediators and mechanisms
Positive feedback loops	Prolong active signaling	Hetero-dimers containing HER2 often evade negative regulation. This is due to production of local EGF-like ligands and angiogenic factors upon activation of receptor
Negative feedback loops	Reduction in number of receptors	Multiple mechanisms involved: post-translational modifications, compartmentalization, catalytic inactivation, and steric hindrance. Pre-existing attenuators primarily control receptor phosphorylation and degradation. For example, density-enhanced phosphatase-1 (DEP-1) dephosphorylates HER1/EGFR
Buffering	Up-regulation/down-regulation	Heat-shock protein-90 (HSP90) is the most significant protein involved. When bound to HER2 it acts as a molecular switch – regulating heterodimer formation, catalytic function, and protein stability

### Single HER inhibitors

Single molecular tyrosine kinase inhibitors (TKIs) are a class of extensively investigated and developed therapeutics that are effective alone and in combination with conventional chemotherapeutics in treating a variety of cancer subtypes. Their mechanism of action is *via* inhibition of the single members of the HER-family culminating in inhibition of downstream signaling (37, 38). More specifically, TKIs such as erlotinib take effect *via* reversible binding to the kinase catalytic domain of EGFR/HER1 preventing auto-phosphorylation and downstream activation (39). Alternatively, mAbs, such as trastuzumab (40) and cetuximab (41), bind to the extra-cellular segment of HER-family members leading to down-regulation.

The success of reversible TKIs has been most marked in patients harboring an *EGFR/HER1* gene mutation that increases TKI sensitivity. Analysis of non-small cell lung cancer (NSCLC) patients has shown that 70–80% of *EGFR/HER1* gene-mutated patients are responsive to TKIs compared with 10–20% of *EGFR/HER1* wild-type patients (42). In contrast, mAbs mode of action varies from those of small molecule inhibitors. Accordingly their inhibitory effects are less dependent upon activation-inducing mutations in the receptor and even downstream changes. This may explain the increased therapeutic impact compared to TKIs on patients with breast and colon cancers (41).

#### Shortcomings of single HER inhibitor treatment and strategies to overcome resistance

Despite successful drug development targeting single HER receptors, patient response has been less than expected, and development of resistance has been a major issue. The primary efficacy of erlotinib and gefitinib in malignancies harboring an *EGFR/HER1* activating mutation is well established. However, this efficacy has shown to be short lived with patients developing secondary mutations conferring drug resistance. These mutations occur at a median time of 12 months from commencement of treatment (42). The *EGFR/HER1 T790M* resistant mutation involves substitution of threonine 790 with methionine, conferring drug resistance by increasing ATP affinity. Inhibition is initially possible due to the compromised ATP affinity resulting from *EGFR/HER1* mutations, and as such, the *T790M* mutation culminates in restoration of wild-type level ATP affinity and a decreased affinity of TKIs targeting HER receptors. The *T790M* mutation is believed to account for 50% of the resistance to the aforementioned drugs (43). Other secondary mutations specific to *EGFR/HER1* resistance development include *D761Y*, *L747S*, and *T854A* (44). However, these have only been reported with low frequency and are not the focus of treatments to overcome resistance. Recently, several third-generation, irreversible, selective EGFR inhibitors such as AZD9291 and CO1686 have shown promise in pre-clinical studies and provided hope for patients with advanced lung cancers that have become resistant to gefitinib or erlotinib. AZD9291 and CO1686 inhibited both the activating (*L858R*) and resistant (*T790M*) *EGFR* mutations in cell culture and in animal models. Conversely, these drugs did not inhibit the wild-type *EGFR* that is present in normal skin and gut cells, thereby reducing the side effects encountered with existing reversible EGFR inhibitors (45–48). Both compounds are undergoing clinical trials and the preliminary data have shown partial responses with good tolerability, according to RECIST (response evaluation criteria in solid tumors), which is a set of published rules that define when cancer patients improve (“respond”), stay the same (“stable”), or worse (“progression”) during treatment (49, 50).

In addition to *EGFR* mutations, *MET* amplification/over-expression was also reported as another common EGFR TKI resistance mechanism in lung cancer. *MET* amplification was observed in 21% of patients with acquired resistance to EGFR TKI (in only 3% of untreated patients) and developed resistance to gefitinib by driving HER3-dependent activation of PI3K in a gefitinib-sensitive lung cancer cell line (HCC827) (6, 7), as well as by activating STAT3 transcription factor directly, through an SH2 domain (51). Targeting MET can enhance EGFR inhibition. EGFR inhibitors combined with MET tyrosine kinase inhibitors such as SU11274 and PHA665752 or MET antibody such as DN-30 synergistically inhibited cell proliferation and promoted apoptosis in NSCLC (52, 53). A number of MET inhibitors are currently in late phase clinical trials (54, 55).

Trastuzumab is a monoclonal antibody against HER2 and was approved for the treatment of HER2-overexpressed metastatic breast cancer in 1998 (16). However, the majority of patients who initially responded to trastuzumab showed partial or complete resistance and disease progression in <1 year post treatment (18). Multiple mechanisms of trastuzumab resistance have been reported (56) including constitutive activation of downstream PI3K/AKT signaling through PTEN down-regulation or PIK3CA hyper-activating mutations, lack of an effective ADCC immune response as well as increased expression or compensatory signaling through other receptor tyrosine kinases (IGF-IR, EGFR, or HER3). IGF-IR signaling (17, 57) was demonstrated to contribute the molecular resistance mechanism to HER2-targeted therapy via cross-talk between IGFR-IR and HER2, which activated the PI3K/AKT and Ras/Raf/MAPK signaling cascades. In addition, trastuzumab only blocks HER2-mediated signaling, but it may not inhibit signaling mediated from other HER receptors (such as EGFR/HER3 or EGFR/EGFR) (18). Therefore, inhibiting multiple HER-family receptors concurrently may be more effective than trastuzumab-based therapy alone.

Resistance to monoclonal antibody (e.g., cetuximab) or small molecule receptor inhibitor (gefitinib or dacomitinib) therapy is most frequently through mutations that result in downstream activation of the pathway eliminating the opportunity for upstream blockade of external signaling; for example, cetuximab blockade to EGFR/HER1. Karapetis et al. (58) in a randomized trial of single agent cetuximab vs. best supportive care showed that a *k-ras* mutation was a predictor for resistance to cetuximab in patients. Drugs that target molecules downstream of k-ras (e.g., b-raf or MEK inhibitors) are needed to effectively manage these patients.

Ciardello et al. (59) have shown that *HER2* gene amplification is another mechanism of resistance to EGFR/HER1 targeted therapy. Their study revealed that resistance to cetuximab in metastatic colorectal cancer “xenopatients” (patient-derived xenografts) was frequently coupled with HER2 over-expression. When treated with EGFR and HER2 inhibition, long lasting tumor regression suggested that panHER inhibition will have clinical benefit in such patients. In addition, blocking of EGFR/HER1 has no impact on the HER2, HER4, and JAK/STAT pathways (by-pass signaling pathways), in which activation of STAT3 is associated with tumor growth and malignancy (5, 60). HER2 acts as a STAT3 co-activator for cyclin D1 promoter activation to promote tumor proliferation (61).

This gives context to the potential benefit of panHER inhibitors. These irreversible TKIs overcome resistance by binding covalently to HER receptors, which inhibits tumor growth in cancers, which are driven by HER heterodimerization and co-expression (62).

## panHER Inhibition Approach

In response to the clinical limitations of EGFR/HER1 TKIs, the role of panHER inhibitors is gaining increased attention. In cancers responsive to HER pathway stimulation, as discussed above, panHER inhibition could provide a means of blocking signaling pathways that are not associated with *EGFR/HER1* activating mutations or gene amplification (hence lower response rates to reversible TKIs). While panHER inhibitor therapy seems to be most effective in patients harboring an activating EGFR mutation, it also shows therapeutic benefit in patients who do not have a HER activating mutation. Pre-clinical studies have shown inhibition of wild-type EGFR/HER1 as well as wild-type HER2 using novel panHER inhibitors (63). Late stage cancers such as lung, breast, head and neck, gastric, and colorectal, which are associated with over-activity of the HER pathways, may all benefit from this new class of drugs (9).

Effects of a range of panHER inhibitors targeting EGFR/HER1, HER2, and HER4 have been explored including AC-480 (formerly known as BMS 599626), HM781-36B, Canertinib (CI-1033), Nertinib (HKI272), afatinib (BIBW2992), and dacomitinib (PF299804). Multi-target TKIs such as lapatinib (an inhibitor of HER1 and HER2) are already approved and in regular use in clinical settings, however, resistance has become apparent *via* escape route signaling (64). A panHER approach demonstrated potential to prevent escape route resistance *via* the JAK/STAT pathway. AC-480 and HM 781-36B have only just reached or completed phase I testing, respectively (65, 66). Canertinib and Nertinib have already entered phase II (67, 68), while dacomitinib and afatinib have been extensively tested in a range of cancer types and are currently undergoing Phase III studies (69). Accordingly, dacomitinib and afatinib will be introduced in the sessions that follow and Tables 3 and 4 include examples of targeting panHER for cancer therapy. Both compounds have two attractive features including irreversible binding to the targeted receptors to extend the efficacy of an agent and overcome resistance and, in addition, broad inhibition of all cancer-relevant HER-family homo-dimers and hetero-dimers to potentially improve efficacy and limit alternative signaling from receptor cross-talk.

**Table 3:** **Clinical trials with dacomitinib**.

	Method	Findings	Tumor type	Reference
2009	Two-arm phase II: efficacy and safety of dacomitinib in patients (pts) after failure of chemotherapy and erlotinib (US)	Stable disease was observed in 9/18 pts in Arm A (adenocarcinoma) and1/2 pts in Arm B (non-adenocarcinoma). Treatment (Tx)-related adverse events (AEs) were skin and gastrointestinal disorders	NSCLC	(70)
2010	Phase I/II: in Korean pts with k-ras wild-type adenocarcinoma NSCLC refractory to chemotherapy and erlotinib or gefitinib	Dacomitinib (*n* = 30) showed 35% progression-free survival rate at 4 months, 87% overall survival rate at 6 months, 8% objective response rate, and 20% clinical benefit rate (partial response or stable disease ≥24 weeks)	NSCLC	(71)
2010	Phase II: efficacy and safety of dacomitinib as first-line treatment of patients with advanced NSCLC selected for activating mutation of EGFR	All evaluable pts with known EGFR-activating mutant NSCLC (*n* = 14) showed tumor shrinkage. Treatment-related adverse event were: diarrhea, dermatitis acneiform, and stomatitis	NSCLC	(72)
2011	Phase I: 121 patients treated with dacomitinib either intermittently or daily continuously	Side effects included diarrhea, rash, fatigue, and nausea. Dacomitinib can be safely administered up to 45 mg/d	NSCLC, colorectal, breast, ovarian, biliary, other	(73)
2012	Phase I: safety and tolerability of dacomitinib in Japanese pts with advanced solid tumors (*n* = 13)	Dacomitinib 45 mg/d was defined as the recommended phase II dose and demonstrated preliminary activity in Japanese pts with advanced solid tumors	Breast, colon, lung, and metastatic neoplasm	(74)
2012	Phase II: observing efficacy of dacomitinib (*n* = 94) vs. erlotinib (*n* = 94) in patients after failure of chemotherapy	Dacomitinib showed significantly longer PFS vs. erlotinib in the overall population (2.86 vs. 1.91 months, *p* = 0.012), with benefit most notable in k-ras wild-type/EGFR any status, k-ras wild-type/EGFR wild-type, and EGFR mutants; and higher objective response rate (17.0 vs. 5.3%, *p* = 0.011). While toxicity was acceptable; treatment-related side adverse effects were more frequent in dacomitinib	NSCLC	(69)
2013	Phase II: clinical activity of dacomitinib as first-line treatment in recurrent and/or metastatic squamous-cell carcinoma of the head and neck (*n* = 69)	In the response-evaluable patients (*n* = 63), 12.7% pts achieved a partial response, 57.1% had stable disease, and 14.3% lasting more than 24 weeks. The median PFS was 12.1 weeks and the median OS was 34.6 weeks. Most AEs were tolerable	Head and neck	(75)

**Table 4:** **Clinical trials with afatinib**.

	Method	Findings	Tumor type	Reference
2008	Phase I: dose-escalation study in 2-week on, 2-week off schedule	Seven patients displayed stable disease lasting more than four cycles. The PK profile absorption showed oral bioavailability was moderately fast, and had a half-life suitable for once-daily dosing	Advanced solid tumors	(76)
2010	Phase I: safety, MTD, and pharmacokinetics of continuous once-daily oral administration	Three patients with NSCLC experienced confirmed partial responses. Seven patients had disease stabilization lasing more than 6 months. PK was a dose-proportional relationship, with reduced drug absorption after food intake	Advanced solid tumors	(77)
2012	Phase I: LUX-lung 4 study in patients with NSCLC after failure of chemotherapy/erlotinib/gefitinib	Six patients had tumor size reduction and three achieved durable stable disease. Peak plasma concentrations were reached 3–4 h after administration with a half-life of 30–40 h at steady-state	Advanced NSCLC	(78)
2013	Phase I: dose-escalation study of continuous once-daily oral treatment	Five patients had stable disease with a median progression – free survival of 111 days. PK revealed no deviation from dose-proportionality and steady-state was reached on day 8	Advanced solid tumors	(79)
2012	Phase I: continuous oral treatment in combination with cisplatin/paclitaxel (*n* = 26) or cisplatin/5-fluorouracil (*n* = 21)	Disease control was observed in 54 and 29% of patients in combination with cisplatin/paclitaxel and cisplatin/5-fluorourcial, respectively. No relevant PK interaction between afatinib and the chemotherapeutic agents	Advanced solid tumors	(80)
2013	Phase I: pulsatile 3-day administration in combination with docetaxel (*n* = 40)	This combination showed 12.5% objective responses and 22.5% durable stable disease. No drug–drug interactions were observed between afatinib and docetaxel	Advanced solid tumors	(81)
2012	Phase II: efficacy of afatinib as first (*n* = 61) or second line treatment (*n* = 68) (LUX-lung2)	66% first-line and 57% second line treatment patients showed objective response after treatment with afatinib daily. The most common adverse events were diarrhea and rash or acne	Advanced lung adenocarcinoma with EGFR mutations	(82)
2012	Exploratory phase II: efficacy of afatinib in patients with HER2 mutations (*n* = 3)	All three patients with activating HER2 mutations in exon 20 showed objective response even after failure of other EGFR- and/or HER2-targeted treatment	Advanced lung adenocarcinoma with HER2 mutations	(83)
2012	Phase II: efficacy and safety of afatinib as second or third-line treatment (*n* = 50)	Afatinib achieved clinical benefit for at least 4 months in a small number (*n* = 3) of heavily pre-treated unselected patients with triple-negative breast cancer. The most common treatment-related adverse events were diarrhea and skin	HER2-negative metastatic breast cancer	(84)
2012	Phase IIb/III: afatinib vs. placebo as third or fourth line treatment in 585 patients (LUX-Lung 1)	Afatinib showed longer progression-free survival (3.3 vs. 1.1 months, *p* < 0.0001) and higher partial response (29 patients vs. 1 patient, *p* < 0.01). There was no significant difference in overall survival between afatinib (10.8 months) and placebo group (12 months, *p* = 0.74)	Advance NSCLC	(85)
2012	Phase III: afatinib vs. pemetrexed and cisplatin as first-line treatment in 345 patients (LUX-Lung 3)	Afatinib showed longer progression-free survival (11.1 vs. 6.9 months, *p* = 0.0004), and higher objective response rate (56 vs. 23%, *p* < 0.0001) than pemetrexed/cisplatin	Advanced lung adenocarcinoma with activating EGFR mutations	(86)
2013	Phase III: safety and efficacy of first-line afatinib vs. gemcitabine/cisplatin in Asian patients with EGFR mutation (LUX-Lung 6) (*n* = 364)	Afatinib showed significantly prolonged progression-free survival (11.0 vs. 5.6 months, *p* < 0.0001), higher objective response (66.9% vs. 23.0%, *p* < 0.0001) and disease control (92.6% vs. 76.2%, *p* < 0.0001) rates, compared with gemcitabine/cisplatin. Overall survival, based on 43% of events showed *p* = 0.7593.	Advanced lung adenocarcinoma with activating EGFR mutations	(87)

### Representative panHER inhibitor: Dacomitinib

Dacomitinib (PF299804) is a second-generation small molecule tyrosine kinase irreversible panHER inhibitor, which is currently involved in Phase III trials for NSCLC and Phase II for Head and Neck cancer (published clinical trials involving dacomitinib to date are summarized in Table 3). It comprises a structurally related quinazoline based core scaffold, as well as an electrophilic motif that covalently binds Cys-797 of EGFR (88). Dacomitinib is potent and highly selective for the EGFR/HER1, HER2, and HER4 members of the HER (erbB) signaling pathway (73). It is believed to irreversibly inhibit HER tyrosine kinase activity through binding at the ATP site and covalent modification of nucleophilic cysteine residues in the catalytic domains of HER-family members.

#### Pre-clinical studies

Dacomitinib is a highly effective inhibitor of *EGFR* activating and *EGFR T790M* acquired resistant mutations as well as the wild-type *HER2*, gefitinib-resistant oncogenic *HER2* mutation, and *HER2* amplification both *in vitro* and *in vivo* in a broad range of human cancer cell lines including lung cancer, gastric cancer, biliary tract cancer, breast cancer, head and neck cancer, ovarian carcinoma, and squamous-cell carcinoma (63, 89–93). Treatment with dacomitinib reduced phosphorylation of HER-family members (EGFR, HER2, and HER4) and downstream AKT and Erk pathways, as well as induced apoptosis and caused G0/G1 arrest (91, 92). Furthermore, excellent pharmacodynamic effects (such as high bioavailability, long half-life, and large volume of distribution) with this compound were observed across species including rats, monkeys, and dogs (93). Despite apparent success in treating tumors with an *EGFR/HER1 T790M* resistant mutation, pre-clinical studies (88) using cell lines with *EGFR T790M* showed that dacomitinib resistance can develop both *in vitro* and using a xenograft model *in vivo* by *EGFR/HER1 T790M* amplification. The activation of IL-6R/JAK1/STAT3 signaling has been identified as a mechanism of *de novo* resistance to irreversible panHER inhibitors in NSCLC with *T790M* resistant mutation (94).

#### Phase I studies

Three Phase I studies in patients with advanced malignant solid tumors in United States, Japan, and South Korea indicated that dacomitinib has attractive pharmacokinetics and metabolism including great bioavailability, long half-life (59–85 h with dosing ranging from 30 to 60 mg), large volume of distribution (2,610 L with the dosing of 45 mg), and low clearance (23.7–32 L/h across the dosing of 15–45 mg) (71, 73, 74). These studies suggested the maximum tolerated dose (MTD) was 45 mg/d, and both continuous and intermittent treatment schedules were well-tolerated. However, this has revealed that concentrations, which are clinically achievable may not allow maximum inhibition of cells harboring the *T790M* resistant mutation. Treatment-related adverse effects (diarrhea, acne, and rash) associated with EGFR/HER1 blocking TKIs were more common in dacomitinib compared with erlotinib (95).

#### Phase II studies

In two subsequent phase II trials in NSCLC as a third-line treatment, dacomitinib was well-tolerated and showed encouraging activity in patients after failure of prior chemotherapy and erlotinib or gefitinib treatment (70, 71). A randomized Phase II trial of 188 patients with advanced NSCLC demonstrated a significant improvement of progression-free survival (PFS) in patients receiving dacomitinib (2.86 months) compared to erlotinib (1.91 months) (69). In two Phase II studies of dacomitinib as a first-line therapy, dacomitinib demonstrated clinical benefits in patients with recurrent and/or metastatic squamous-cell carcinoma of the head and neck (RM-SCCHN) and advanced NSCLC (72, 75).

Dacomitinib, currently under phase III studies (JBR-26 and NCT01360554), appears to be an example of how the panHER approach to targeted therapy may hold promise for improving targeted treatment outcomes. Activity demonstrated in NSCLC may also have an application within other cancer types with positive HER-family expression such as other epithelial cancers (96) and sarcoma (97).

### Representative panHER inhibitor: Afatinib

Afatinib, an aniline–quinazoline derivative with a functional Michael acceptor group, is designed to covalently bind the catalytic domains of EGFR (Cys-797), HER2 (Cys805), and HER4 (Cys803) and irreversibly block enzymatically active HER-family members (98).

#### Pre-clinical studies

In cell-free *in vitro* kinase assays, afatinib potently inhibited wild-type *EGFR* (EC_50_ = 0.5 nM), HER2 (14 nM), and HER4 (1 nM), as well as oncogenic *L858R* activating mutant *EGFR* (0.4 nM) and *L858R-T790M* resistant mutant *EGFR* (10 nM) (98, 99). Afatinib inhibited auto-phosphorylation of HER members and proliferation in cancer cell lines representing different mutational status of *EGFR* and *HER2*, including wild-type *EGFR* (A431), activating mutant *EGFR* (PC-9 and H3255, EC_50_ = 0.4 and 0.5 nM, respectively), *L858R-T790M* mutant *EGFR* (NCI-H1975), L858R-T854A mutant EGFR (transfected 293T), and *HER2* amplification (BT474) with EC_50_ below 100 nM, whereas NCI-H1975, T854A transfected 293T, and BT474 were resistant to erlotinib and/or gefitinib (98, 100). Furthermore, afatinib induced tumor regression in a broad spectrum of xenograft models carrying epidermoid carcinoma A431 with wild-type *EGFR*, gastric cancer NCI-N87 with HER2 over-expression, lung cancer NCI-H1975 with *L858R-T790M* mutant *EGFR*, and adenosquamous lung tumor with *HER2^YVMA^* mutation, with superior activity over erlotinib (99, 101). In a panel of seven human pancreatic tumor cell lines, afatinib indicated greater efficacy in antiproliferation and signaling blockage (phosphorylation of EGFR, MAPK, and AKT) (102). *In vivo*, daily administration of 15 mg/kg afatinib showed potent antitumor activity in the BxPC-3 human pancreatic xenograft model (102).

Clinical trials using Afatinib are listed in Table 4.

#### Phase I studies

Several Phase I dose-escalation studies with afatinib indicated that afatinib is well-tolerated (76–79, 103). The recommended dose in 2-week on, 2-week off schedule is 70 mg/d (76), while in once-daily oral treatment is 40–50 mg/d in advanced solid tumors and in advanced NSCLC after failure of chemotherapy/erlotinib/gefitinib (LUX-Lung4) (77–79). The most frequent treatment-related adverse events were diarrhea, mucosal inflammation, and skin rash. A pharmacokinetic study in healthy male volunteers with afatinib showed the main route of excretion is *via* feces, and it undergoes minimal metabolism (103).

#### Phase II studies

LUX-Lung 2, a single-arm phase II study in 129 patients with lung adenocarcinoma containing activating *EGFR* mutations within exons 18-21, showed antitumor activity after treatment as first or second line with afatinib daily 40 or 50 mg (82). Afatinib was more effective (objective response: 66%) in patients with the common *EGFR* mutations (*deletion 19* and *L858R*) compared to patients with less common mutations (39%) (82). In addition, afatinib indicated therapeutic activity in three patients with lung adenocarcinoma and a non-smoking history, whose tumors exhibited activating *HER2* mutation in exon 20 (83). However, afatinib had limited activity in HER2-negative breast cancer (84). None of the fifty patients achieved an objective response, while three patients with triple-negative metastatic breast cancer had stable disease for more than four treatment courses. This indicated that further examination of afatinib in patients should select population with *EGFR/HER2* mutations or HER receptor/ligand over-expression or activation to increase the likelihood of a meaningful clinical benefit.

#### Phase III studies

LUX-Lung 1 was a randomized, double-blind phase IIb/III study comparing afatinib with placebo in 585 NSCLC patients after failure of chemotherapy/erlotinib/gefitinib (85). Afatinib failed to show a difference between groups at its primary endpoint and overall survival, although the PFS findings were promising. One of the potential reasons is that the number of patients with EGFR mutations was unknown, since EGFR mutation status was not required for study entry. Accordingly, LUX-Lung 3 examined the efficacy of afatinib compared with pemetrexed and cisplatin as first-line treatment for patients with advanced lung adenocarcinoma harboring *EGFR*-activating mutations. Encouragingly, afatinib led to a prolonged PFS, more significantly with common *Del19* and *L858R* mutations (86, 87).

In July 2013, afatinib was approved for the first-line treatment of patients with metastatic NSCLC with *EGFR* exon 19 deletions or exon 21 (*L858R*) substitution mutations by the US Food and Drug Administration (104).

### panHER combination therapy

Combination therapy involving panHER inhibitors is also under investigation. This varies from combinations of targeted therapies with either radiation or chemotherapy or combinations of targeted therapies directed against different aspects of the EGFR pathway.

Following radiotherapy, tissue recovery is associated with increased generation of HER ligands to promote the survival and cell proliferation that HER signaling provides. One potential outcome of this tissue repair event is tumor recurrence in patients with high HER expression. It follows biologically that blocking HER pathways simultaneously may impair this process and thus enhance radiation effectiveness (105). Torres et al. (106) showed that panHER inhibitor AC-480 increased tumor growth delay (enhancement factor = 1.94) when used in combination with radiotherapy in head and neck squamous-cell carcinoma in mice.

panHER inhibitors in combination with chemotherapy have successfully showed synergistic results in both pre-clinical studies and clinical trials. Combination therapy in NSCLC cells harboring a *T790M* mutation both *in vitro* and *in vivo* showed (107) that gefitinib (EGFR/HER1 TKI) used in combination with antimetabolites, either fluorouracil (5FU) or pemetrexed, had an antagonistic growth inhibitory effect *in vitro*. In contrast, afatinib both *in vitro* and *in vivo* was synergistic with these drugs. In two phase I studies of afatinib combination with chemotherapy, the MTD for afatinib was 20 mg with cisplatin/paclitaxel, 30 mg with cisplatin/5-fluorouracil, and 90 mg for 3 days after docetaxel in advanced solid tumors (80, 81). Both studies showed antitumor activities with a manageable adverse-event profile. CI-1033 is another panHER inhibitor with promising lab data showing it synergizes with cisplatin *in vitro* (108). This is believed to be because cisplatin inhibits key genes in cell survival when the EGFR pathway is blocked simultaneously. CI-1033 has undergone a phase I trial in combination with docetaxel in advanced solid tumor patients. It appeared to be safe with acceptable side effects following an intermittent administration schedule (109). CI-1033 has also been examined in combination with paclitaxel and carboplatin in advanced NSCLC patients (110). Phase I studies showed it was well-tolerated as well as having possible synergistic antitumor trends, which await confirmation in phase II studies. Lapatinib (HER1/HER2 TKI) has been approved by the FDA for routine clinical use in combination with capecitabine (prodrug of 5FU). This is on the basis of phase III studies showing improved PFS in HER2 positive breast cancer (12.0 weeks in combination therapy vs. 8.4 weeks in lapatinib alone) (111).

Another approach to target the HER-family is through concurrent treatment with new bispecific antibodies such as MM-111 and an HER2 antibody or small molecular inhibitor. MM-111 targets the HER2/HER3 heterodimer with specificity and avidity by docking onto HER2 and subsequently binding to HER3 and blocking Heregulin-induced activation of HER3, therefore, it is more effective at inhibiting HER3 activation than a HER2 or HER3 monoclonal antibody (trastuzumab and pertuzumab) and TKI (lapatinib) (112). In addition, combining MM-111 with other HER2-targeted agents (trastuzumab and/or lapatinib) and chemotherapy (such as paclitaxel) synergistically inhibit tumor cell growth and prevent the development of HER3-driven drug resistance in gastric and breast cancer cells and xenografts (113–115). Currently, a Phase II clinical trial combining MM-111 with paclitaxel and trastuzumab is being investigate (116).

The development of bispecific antibodies, along with the third-generation irreversible small molecular inhibitors, have improved the efficacy to target HER-family, even though they clearly differ in their mode of action at target level: for example, MM-111, consisting of fully human anti-HER2 and anti-HER3 single chain antibody moieties linked by modified human serum albumin, targets the extra-cellular domain of HER2/HER3 heterodimer and promotes the formation of inactive trimetric complexes. In comparison, the third-generation EGFR TKIs (AZD9291 and CO1686) act on the intracellular ATP binding domain and irreversibly inhibit both the activating and resistant *EGFR* mutations (45, 48, 112). Based on our current knowledge, there is no apparent distinction between antibodies and TKIs regarding HER-family targeting effects. However, their differences could be exploited in certain clinical situations. For example, patients with activating and/or resistant *EGFR* mutations should be considered for the use of third-generation EGFR TKIs, whereas patients with over-expression of HER2/HER3 should be treated by bispecific antibody MM-111. Generally, TKIs as small molecules are able to penetrate the blood-brain barrier, indicating that these agents may be therapeutic in patients with primary or metastatic central nervous system disease (117). By contrast more clarity about the potential for ADCC to have a role in antibody-mediated antitumor activity may encourage development of this as the favored therapeutic approach. Especially FcγR genotype may be a predictor of efficacy (118). Indeed rather than focusing on identifying which approach is optimal, it is possible that inhibition of multiple sites of HER receptor activation using both antibody and TKI will prove to be the best way forward. A recent example showed an enhanced antitumor activity when HER-antibody trastuzumab was combined with an EGFR TKI gefitinib or erlotinib (117).

### Soft tissue sarcomas as a novel target

EGFR/HER1 is frequently overexpressed in soft tissue sarcoma, with 78% of patient tissue samples showing positive EGFR/HER1 expression in a study by Yang et al. (119). Early data suggest that blocking EGFR/HER1 by its specific inhibitor may have activity in sarcomas through tyrosine kinase signaling inhibition (14). However, such blockade has no impact on the HER2, HER4, and JAK/STAT pathways, in which activation of STAT3 is associated with tumor growth (120). This problem with single receptor blockage is also confirmed in clinical studies (121), showing that erlotinib was not active in malignant peripheral nerve sheath tumors (MPNST), which are relatively resistant to chemotherapy. Similarly, cetuximab has minimal activity in EGFR positive advanced sarcoma (122).

Recently, a phase II trial in osteosarcoma patients with HER2 over-expression showed that blocking HER2 using trastuzumab in combination with cytotoxic chemotherapy had no clinical benefit (123). The promising data discussed earlier regarding panHER combination therapy could justify a trial in such disease groups to overcome the failure of single targeting.

## Conclusion

Targeted therapy against the HER-family individual members or panHER-family has shown potential to improve prognosis in sensitive patients in some tumor types. One mechanism of resistance to EGFR/HER1 therapies is HER2, 3, 4, and JAK/STAT by-pass signaling. Pre-clinical studies and clinical trials of panHER inhibitors show promising results, and the potential to improve patient outcomes in NSCLC and head and neck cancers. In some cancer patients, the panHER inhibition approach met resistance from HER-independent JAK/STAT by-pass signaling. The use of STAT3 blockage in combination with panHER inhibition in cancers including epithelial cancers and sarcoma, with HER-family hyper-activation and resistance to panHER inhibitor, is a new direction to explore and has potential in clinical cancer therapy in the future.

## Conflict of Interest Statement

The authors declare that the research was conducted in the absence of any commercial or financial relationships that could be construed as a potential conflict of interest.

## Abbreviations

EGFR, the epidermal growth factor receptor; GRB2, growth-factor-receptor bound-2; HER, the human epidermal growth factor receptor; JAK/STAT, the Janus kinas/signal transducer and activator of transcription; MAPK, the mitogen activated protein kinase; MEK, mitogen activated protein kinase kinase; MTD, maximum tolerated dose; mTOR, mammalian target of rapamycin; NSCLC, non-small cell lung cancer; PI3K/AKT, the phosphoinositide 3-kinase/AKT; PKB, protein kinase B; SHC, Src-homology-2-containing; TKD, tyrosine kinase domain; TKIs, tyrosine kinase inhibitors.
